# Niche construction, social cognition, and language: hypothesizing the human as the production of place

**DOI:** 10.1007/s40167-016-0039-2

**Published:** 2016-09-03

**Authors:** Oliver Davies

**Affiliations:** Department of Theology and Religious Studies, King’s College London, Virginia Woolf Building, 22 Kingsway, London, WC2B 6LE UK

**Keywords:** Enactivism, Social cognition, Language evolution, Place, Religion, Niche construction

## Abstract

New data is emerging from evolutionary anthropology and the neuroscience of social cognition on our species-specific hyper-cooperation (HC). This paper attempts an integration of third-person archaeological and second-person, neuroscientific perspectives on the structure of HC, through a post-Ricoeurian development in hermeneutical phenomenology. We argue for the relatively late evolution of advanced linguistic consciousness (ALC) (Hiscock in Biological Theory 9:27–41, 2014), as a reflexive system based on the ‘in-between’ or ‘cognitive system’ as reported by Vogeley et al. (in: Interdisziplinäre anthropologie, Heidelberg, Springer, 2014) of face-to-face social cognition, as well as tool use. The possibility of a positive or negative tension between the more recent ALC and the more ancient, pre-thematic, self-organizing ‘in-between’ frames an ‘internal’ niche construction. This indexes the internal structure of HC as ‘convergence’, where complex, engaged, social reasoning in ALC mirrors the cognitive structure of the pre-thematic ‘in-between’, extending the bio-energy of our social cognition, through reflexive amplification, in the production of ‘social place’ as ‘humanized space’. If individual word/phrase acquisition, in contextual actuality, is the distinctive feature of human language (Hurford in European Reviews 12:551–565, 2004), then human language is a hyperbolic, species-wide training in particularized co-location, developing consciousness of a shared world. The humanization of space and production of HC, through co-location, requires the ‘disarming’ of language as a medium of control, and a foregrounding of the materiality of the sign. The production of ‘hyper-place’ as solidarity beyond the face-to-face, typical of world religions, becomes possible where internal niche construction as convergence with the ‘in-between’ (world in us) combines with religious cosmologies reflecting an external ‘cosmic’ niche construction (world outside us).

## Preface

The dialogue between science and the humanities is on-going, and remains vital in modern society. But it easy to take the view that two influential ways of thinking about the human which have emerged in recent years are of unusual significance and so potentially of broader relevance to society. The first is archaeological and is an evolutionary account of the development of distinctive traits of our basal sociality as hyper-cooperation (HC) in genus *Homo* extending for at least around two million years into our hominin past. The second is neuroscientific and concerns recent breakthroughs in our understanding of ‘live’ social cognition. We are an evolved, social creature, and these methodologically distinct but related accounts can potentially cast light on who we are today, from the perspective of our foundational sociality, in a changing world.

But understanding what this new knowledge *means*, in a way that can inform our understanding of present social and political realities, calls for a multi-layered, hermeneutical philosophy with a philosophical perspective which includes linguistic-literary theory, on the one hand, and historical philosophy, or theology, on the other. This philosophy will be required to integrate archaeology and neuroscience around an enriched human centre which includes temporality, intentionality and our human particularity or actuality: our capacity to be *this* person at *this* place and time. Linguistic-literary theory points to the fecund specificity of human language, nowhere more evident than in the impossibility of exact translation between words: *traduttore*–*traditore*. But it will also need to be able to access conceptualities of the human person, and of language, from different historical periods, including the pre-modern anthropologies which share the non-dualism of our contemporary neuroscientific accounts of the self and which, in their theological format, can reflect a more participative account of the self. Western Christianity also carries a unique memory of cosmological change in the ‘first’ scientific revolution of the early modern period, with its extensive recalibration of matter. Pre-modern conceptualisations of the human can also be important resources for expanding our contemporary self-understanding since, arguably, both modern and pre-modern accounts of person and language are equidistant from the significant advances in the science of today.^1^

## Introduction

The archaeology of HC and the neuroscience of its internal structure are separated by the former’s ‘third-person’ optic and distancing focus on the past and by the latter’s ‘second person’ focus on the ‘live’ or ‘online’ present of human encounter, together with its clinical orientation in autism studies. The potential for each to learn from the other calls for a more formal philosophical mediation, in the generation of terms which can allow each to come into focus with respect to the other, in the context of integrating our understanding of the human today.

But notably both the archaeology of HC and the neuroscience of its internal structure have one significant feature in common: language remains stubbornly liminal. Our social cognition sits primarily within the pre-thematic reflexes of the motor system, and the anthropologist can happily bond with her higher primates who don’t share her advanced linguistic consciousness (ALC), as she can with human infants, or linguistically impaired adults. Indeed, it may be easier to bond with the non-linguistic rather than the linguistic other. On the other hand, the evolution of language has been called ‘the hardest problem in science’ (Christiansen and Kirby 2003, pp. 1–15), involving multiple social, environmental, neurological and cognitive factors, as these extend individually into the remote past, but also as these factors may be capable of sudden interaction in new ways (Fitch 2010). The evidence for cognition extends further and further into our hominin past, while tool-use, especially Levallois tool-use, and the increasing communicative and structural complexity of larger-scale hunter-gatherer societies insistently suggests that the emergence of ALC from proto-language is relatively recent, dating from around 100,000 years ago, or even much later (Dunbar 1996; Wynn 2012; Dor et al. 2014). Perhaps, in fact, it is only at the end of the Paleolithic, in the gregarious townships of the Neolithic with their first full representations of the human face (Kuijt 2008), when territorialization and conflict are on the rise and writing is already on the horizon, that we unequivocally feel that we can see our own linguistic image (Knight and Power 2012; Bickerton 2009; Suzuki et al. 2012). While for ALC language is self-evidently linked with our evolved social cognition, our visceral and mimetic social bonding which underlies HC may not in fact depend upon language. It appears too that our modern language can disrupt or disfigure our hyper-cooperation, or ‘get in the way’, as easily as it can enhance it. Vittorio Gallese has pointed out that the ability to deny another’s humanity is ‘probably one of the worst spin-offs of language’ (All too Human 2014).

Is it the case then that we see language everywhere as ALC, or subjects, when in fact our basal sociality is pre-linguistic? If such a gap or distanciation is the case, then this would suggest that any advance in human self-understanding on the grounds of the interaction of HC and social cognition will rest on the *relation* between language and our basal sociality, calling for a particular kind of critical, non-dualist philosophy. But what kind of philosophy? The ‘appearance’ of this new knowledge of the internal structure of HC in the display of fMRI data, suggests that we can place our inquiry in the field of phenomenology, which was classically defined by Martin Heidegger in its concern with sense-making and letting ‘that which shows itself be seen’ (Heidegger 1962, p. 58). But in its favoring of meaning over knowledge, and its focus on the role of language, we should think in terms of a distinctive phenomenological hermeneutics.^2^ Arguably this is a tradition which begins in Kant’s late work *Anthropology from a Pragmatic Perspective*, in which he laments that we do not understand ‘the cranial nerves and fibres’ of our physiology (Kant 1798/2006: 3). In this, his final published work, Kant turned to a physicalist understanding of language, and indeed of reason, under the influence of Hamann and Herder (Forster 2012), which inaugurated a Continental philosophical trajectory that led through Merleau-Ponty to the more political, critical philosophy of Michel Foucault, Foucault was himself a keen student of Kant’s *Anthropology* (Foucault 2008). If an innovative phenomenological hermeneutics can internalize the new scientific knowledge of our primary sociality, then it may also take on something of the character of the ‘moral thought’ of eighteenth century Humean tradition, which Kant shared, where ‘moral’ points to accounts of the human which capture something of our capacity for personal wholeness, fullness and actuality (Millican 2002, p. 32). And we may need to take note of German Romantic insights in response to the ‘psychological Kant’ of the three Critiques. Schelling and Schiller emphasized the *aesthetic* properties of human, communicative rationality through the body (glossed as ‘freedom’), which operates transformatively within matter, rather than from a supposed point outside it (Beiser 2005; Schindler 2012).

### Self-naming

We need to begin then with a philosophical determination of who we are, or with an act of self-naming, in the light of new disclosive appearances about our social human nature. Generally self-naming is a form of definition which closes down the myriad possibilities of the human, and fixes us as being this rather than that (Chinese or American, Liberal or Neo-Conservative). A phenomenological hermeneutics which is attentive to understanding the meaning of new appearances of such a fundamental kind, needs in contrast to be both inclusive and integrative. It needs to be physicalist and non-reductive: open to science but also to the humanities. But above all, such a self-naming has to be grounded in our ordinary reality. It needs to be able to capture our human ‘particularity’ which is to say our actuality and simplicity as being *this* human being at *this* place and time. We can associate our ‘particularity’ with the distinctiveness of the human voice and what, on occasion, we have to say, and how we say it. But it also underlies our ‘individuality’ and capacity to act and think ‘responsibly’, as it does our ‘identifiability’ and ‘recognizability’ as such. In this elemental sense, it is the bedrock of a humanistic approach to human personhood, life and culture (cf. Gerard Manley Hopkins: ‘All things counter, original, spare, strange’, ‘Pied Beauty’, Hopkins 2009, p. 132). We shall argue here that our species-wide awareness of being differently located in the world may be the deepest anthropological constant of all and, as we shall argue, it may be that it is in the *realization* of our own locality that our sense of world as *shared* space or place is most strongly generated.

Particularity, which indicates an incontrovertible *complexity*, is not at home in scientific discourse, although every clinician knows the particularity of the patient in the complex unity of mind, body and environment. Alain Badiou warns against ‘an abstract homogenization’ and points instead to the ‘uncountable infinity constituted by a single human life’ which disrupts the logic of economy and statistics (Badiou 2003, pp. 9, 10). Scientific research cannot easily accommodate the particular, since the scientific method maximally reduces complexity in order to gain the secure knowledge it needs. Humanities disciplines tolerate more complexity in their interpretative schemes, and so gain understanding which is certainly less reliable but also more inclusive; and closer to the unpredictability, originality and creativity that we also associate with human particularity. The representations of our particularity in Western art and philosophy can be an important resource therefore for an integrative, science-based philosophy of the human as social, which accepts its foundations in the data of empirical science but also seeks to accommodate rather than by-pass the human particular.

A key early philosophical development with respect to particularity occurs in the work of the medieval philosopher Duns Scotus with his metaphysical concept of ‘thisness’ (*haecceitas*), denoting the concrete actuality and singularity of entities. Scotus seems to link this term with an ethical sensibility and our own higher functions of resilient freedom (Lee 2002, pp. 59–73). The modern French philosopher Gilles Deleuze, recalling Charles Dickens’ account of a dying man who is naked and stripped of all narratives, borrows Scotus’ ‘thisness’ but also calls our human *singularité* ‘the pure immanence’ which ‘is A LIFE, and nothing else. It is not immanent to something but the immanence which is in nothing is itself a life’ (Deleuze 1997, p. 4). This evokes, for Deleuze, a level of existing in the world which is more fundamental than the ‘transcendental’ realm of the cogito and its perceived objects. Deleuze too identifies our recognition of another’s particularity as an ethical moment, with its empathy and compassion. There are cultural parallels in the French poet Paul Valéry’s understanding of actuality in the classical dancer, whose body ‘is both mover and moved’ and which displays ‘a kind of *inner life* […] consisting entirely in sensations of time and energy which respond to one another and form a kind of closed circle of resonance’ (Valéry 1964b, pp. 203, 207). In addition to the body we are, the body others see and the medical body we dissect, Valéry sees a fourth, fundamental body which—in the unity of body and mind—might equally be called both ‘real’ and ‘imaginary’. For Valéry, this belongs to the ‘physicists’, though they don’t yet understand it (Valéry 1964a, pp. 35–40). The modern painter-sculptor, Alberto Giacometti, repeatedly sought to reproduce the ‘pure presence’ of human embodiment in the ‘non-individual’ forms of his elongated human figures, with recognizable but statuesque features. Here the human being is a ‘sign consumed by the power of space’ (Hüttlinger 1967). The particularity of the human appears for Giacometti in the tension between space and personhood, while in Valéry, it appears in the tension between space and movement: the dancer’s grace. And finally we should mention the twentieth century Russian philosopher, Mikhail Bakhtin, who, in his study *Towards a Philosophy of the Act*, argues for the primacy of the *irreversible* actuality of the concrete act (such as signing a contract dated and witnessed), as a testing-ground for the fluidity of culture and the point of its self-interrogation (Bakhtin 1993).

## Niche construction

### Niche construction: external place

The Deleuzian concept of immanence or life, allows us to read particularity in a distinctively physicalist way, as combining both ‘locality’ and ‘actuality’. We are at once ‘local’ and ‘actual’, where the ‘local’ is our locatedness and the ‘actual’ is our power of non-random movement, which is the classifier of life (Odling-Smee and Laland 2009; Godfrey-Smith 1996). We are *locality*-*actuality*, and whatever is distinctive or particular to us lies in the tension between the two. The term ‘locality’ may denote either non-human ‘space’ or humanized ‘place’, while ‘actuality’ is the life-force which determines that distinction. As social creatures, our ‘actuality’ is always reciprocally structured, through thematic and pre-thematic kinetic responses, through the interactivity of our intentional practices and acts, or through shared narratives, imagining or performance. It is our ‘actuality’ which defines whether the ‘locality’ we are, and where we are, is humanized ‘place’, or whether it is non-human ‘space’, which awaits or resists discovery, or domestication, limiting or even threatening place. This is a resistant but ultimately non-stable boundary since ‘space’ can always erupt *within* ‘place’, as the illness, frailty, contingency or mortality of the human body for instance, or as a planetary threat within our environment, or as an unforeseen act of nature, so dislodging us either internally or externally. It can manifest also, arguably as intra-human violence, within the social order. The meaning of ‘space’, repeatedly erupting in contexts of ‘place’, will call to be ‘discovered’, or brought into our frameworks of personal and social meaning.

This kinetic, physicalist reading of the self as particular, potentially sits within the contemporary evolutionary theory of niche construction, and it is the purpose of this paper to reflect upon what kind of contribution to our human self-understanding this refinement might make. Niche construction has been a significant revisory tendency within evolutionary theory since the publication of *Niche construction: the neglected process in evolution* in 2003 (Odling-Smee et al. 2003). It sets the shaping of ‘niche’ (as ‘fitness’ or ‘at-homeness’ in the environment), and so also the concept of ‘place’, at the centre of the evolutionary process. The ‘extended evolutionary synthesis’ (EES), which supports niche construction, can be defined in the following way:

The EES is thus characterized by the central role of the organism in the evolutionary process, and by the view that the direction of evolution does not depend on selection alone, and need not start with mutation. The resulting network of processes provides a considerably more complex account of evolutionary mechanisms than traditionally recognized (Laland et al. 2015, p. 8).

Further:

Niche construction extends contemporary evolutionary theory by the introduction of two innovations. First niche construction assigns a second role to phenotypes in evolution, while ecological inheritance provides a second inheritance system to which phenotypes can potentially contribute. […] There is no requirement for niche construction to result directly from genetic variation before it can influence the selection of genetic variation (Odling-Smee et al. 2003, p. 27).

Niche construction, or the formation of a viable environment for living creatures, shows behavioral responses (constructive development) and interactive behaviors with other members of the species (reciprocal causation) (Laland et al. 2015, p. 6). In the case of genus *Homo*, the construction of our human niche is distinctively characterized by the development of high levels of cooperation and so also of socialization. Our human HC shows the influence of ‘lengthy childhood and complex parenting; intricate and diverse foraging and hunting patterns; novel and dynamic material and symbolic cultures; and complex communication and information sharing, eventually resulting in language’ (Fuentes 2015, p. 302). Extended and intensified sociality is here inscribed as a primary behavioral motor in human evolutionary development, within specific environments. HC and the societal configurations which HC supports, have been an essential element in our species survival, within natural selection.

An immanentist, physicalist focus upon the self, in the light of our particularity, as *locality*-*actuality* offers two particular resources to EES and niche construction. In the first place, a distinction between ‘place’ and ‘space’ is operative across the boundary of the human body: these terms can apply equally to our internal sense of body and mobility, for instance, and to our external experience of purposeful movement in an environment which we seek to shape so that we can be ‘at home’. Arguably this creates the possibility of an enhanced or enriched account of human niche construction as potentially including internal, intuitive or existential dimensions of human experience as well as its external and observable ones. But secondly, against the background of our HC, *locality*-*actuality* can also be said to open up new, critical understandings of the underlying structures which support, sustain and produce hyper-cooperative behaviors. In the dyad, the first term *locality*- opens up the recognition that as human beings we are differently located in a shared world. This shared sense of our being differently located grounds our mutual recognition and supports our strong sociality, accommodating the particularity or unpredictable, dynamic otherness of the other. There is within *locality*- an excess, beyond the limits of the *actuality* of the self, which can be named ‘place’, and which can be said to constitute the possibility of a shared world. In the following sections of this paper, we will seek to explore the productive conditions of this state of a ‘shared world’, in terms specifically of language and the production of ‘social place’ and ‘hyper-place’, as offering an internal mapping of HC.

#### Niche construction and language

It has been argued that language too belongs behaviorally to the evolution of HC within niche construction, as an extension of our ‘non-random’ behavior (Odling-Smee and Laland 2009; Clark 2006; Sinha 2009). The functionality of language as naming is a key mechanism for turning ‘space’ into ‘place’, through processes of internalization and intentionalization.^3^ Naming implies a community who name, for whom naming is reference, or pointing to a world held in common (children learn reference very early in their development) (Tomasello 1998). The capacity *imaginatively* to refer to a world held in common is also key to our HC with its common goals (Fuentes 2014). Language belongs to ‘communicative niche construction’, as the passing down of encoded information in material form across generations, constructing inheritable cultures which can endure far beyond the life-time of the individual and which are themselves operative in the shaping of our environment. It has multiple ancient origins, which include primate calls (Nóbrega and Miyagawa 2015), lip-smacking (Ghazanfar et al. 2012), mutual grooming (Dunbar 1996) and dexterity (Cartmill et al. 2012; Steele et al. 2012), as well as our capacity to produce musical sounds (Masataka 2009) and to dance (Hagendoorn 2010). These origins place language within the ‘face-to-face’ of our social bonding, or what Sloterdijk calls the ‘species-wide, interfacial, greenhouse effect’ (Dor et al. 2014; Sloterdijk 2011: 169).

But there is a second history of language which begins remotely with ancient histories of Oldowan and Acheulean tool-use. More proximately, it can be seen in the ‘lithic landscapes’ of Mousterian and Levallois flint-knapping cultures, from around 70,000 years ago in the Upper Pleistocene, when we begin to see evidence for ‘amodality’ or the transferability of tools across contexts which is such a marked feature of words (which can always be applied in different contexts) (Hiscock 2014; Clark 2011, pp. 44–60). The preparation of transportable flint cores suggests new levels of dexterity and precision, in parallel with the percussion of the strike, but also temporal distention which we associate with stronger forms of memory and shared attentiveness to longer term goals. The ‘lithic’ landscapes of the times suggest that high value tool production may have been public and may have included dimensions of apprenticeship, with its associated additional communicative demands (Hiscock 2014).

Words, like highly worked flints, are transgenerational informational artefacts with which we have learned productively to shape our environments, through forms of symbolic exchange, inheritance and action (Fuentes 2015). But it is arguable that we should think of words both in communitarian terms as the product of the inter-facial constant, and as the product of our more outward facing tool use. Indeed, words are social, inter-personal tools for shaping relationships, underpinning and extending HC. We use words to function together, cooperatively, and for the identification and pursuit of common goals, in the construction of our human niche. Words and tools are parallel but also at times intersecting communicative artefacts, as we see in our contemporary communications technology, generating further more complex, interactive ecologies. But it is evident too that, as outward facing, words can also be used to break down or destabilize our human ‘niche’; like tools, they can be used to slash and hurt and to drive away. They can function as ‘de-humanization markers’ for discrete groups who we judge to be our enemies. As ‘tools of meaning’, words can be used to deceive and mislead, so affecting our understanding of what is constructive or destructive for the viability of our human niche. Our HC can be as much corrupted as it is fostered by our use of words. Positively, words can be used to create cultures of reciprocal particularity, allowing us to express and recognize each other’s ‘locality’ by responsibly elaborating a distinctive point of view within our community and by recognizing the point of view of others. But words can also be used repressively, through ideological motivations or greed, to ‘de-particularize’ or homogenize the checkered fabric of our public and political common life.

#### Objectifying language within niche construction

Language lies at the heart of our contemporary consciousness and is at the centre of our capacities for HC, though also its de-formations. It is important therefore that we gain a maximally objective and critical understanding of *what language is* if we are to place it within niche construction. But there are significant obstacles to this. How do we objectify language, for instance, when it is language itself which is our primary mode of objectification? How can we view language neutrally when we are all language users, often in different ways: the computer programmer, the parent, the poet, the sales person. The history of the philosophy of language suggests that we have understood language in very contrasting ways in different historical periods, but principally harnessed to the dominant philosophies of mind of the day (Davies 2015). Furthermore, it is a fundamental principle of linguistics that communication is determined as much by the words we choose *not* to use, since ‘parole’ or utterance always presupposes a moment of selection within ‘langue’ or language as system. The lexical item we use entails the communicative non-selection of others (de Saussure 1986, pp. 9–10, 15). While the materiality of words supports their objectification, their inherently elective character does not. How can we objectively evaluate the subjectivity of choice that is intrinsic to ALC? Finally, ALC is bound up with the ‘semantic system’ in the neocortex and appears as a late developing adaptation of an expanding human brain, like a membrane or porous, global interface with the world which now presses more richly upon us (Huth et al. 2016). Words allowed us to reduce and manage the world’s complexity through prediction, naming, ordering and hierarchalization (Frith 2007). But the ‘porosity’ of language means that the world can also ‘break into’ consciousness in unpredictable and creative ways, not least through narrative, imagination and art (Williams 2014). This ‘global’ aspect of language again resists objectification. As Andy Clark has rightly indicated, for all their centrality in our human systems of ‘thought and reason’, words ‘remain surprisingly ill-understood’ (Clark 2006, p. 370).

But as Clark himself proposes, a good place to start is with physicalism and the recent scientific insights into the thoroughly material nature of words. These are themselves ‘material objects’ that ‘press minds like ours from the biological flux’ (Clark 2011, pp. 44–60). In their tool-like nature, they are ‘potent real-world structures’ (of sound or shape) which ground the ‘neural wet-ware’ of consciousness, helping us to consolidate and objectify through material form what it is that we think (Clark 2011, p. 56). It is the internalized materiality of the sign which allows the human mind to perform precise mathematical tasks (Clark 2006; Dehaene et al. 1999). And, importantly, this same capacity for sequencing, through material language, is reflected in the way that words allow us to place ourselves, as both mind and matter, in time (Hawkins 2004; Cann et al. 2005; Bakhtin 1993). It is this possibility of our self-placing in the ceaseless flow of time, through the materiality of words, which points also to our capacity to sustain long-term identity and with that the responsibility and inimitability we can associate with human particularity (Williams 2014). From the perspective of the human niche, the advent of advanced language already marks a deeper entry into our material environment on the one hand and its productive re-formation through mind on the other. At this stage of course, this is ‘behavioural’ in a non-reflective sense; it is an evolutionary affordance which lays the ground for reflective design (Withagen and van Wermeskerken 2010; see also Kinsella 2009).

In the search for what is distinctive in human language, and so in ALC, the theme of sequencing, as ‘recursion’, has been prominent. ‘Recursive functions’ take their last output as their next input, generating what the nineteenth century linguist von Humboldt, referred to as ‘the infinite use of finite means’ (Fitch 2010, p. 90). Recursion has been viewed principally as a feature of mathematical and computer languages (Hauser et al. 2002), though recursion as non-identical repetition takes on an added significance in natural languages where the contextual and behavioral ‘meanings’ of recursive functions add real semantic density. As Fitch argues, recursion in natural languages ‘refers to a recursive mapping between signals and meanings’ which ‘we can test for behaviorally in our own species’ (Fitch 2010, pp. 81–83).

As a system of syntactical relations, recursion has an unclear relation to ‘locality’ however, in the *locality*-*actuality* dyad. It appears to be predominantly introspective in the sense that it is denotes a movement which can be taken to be internal to mind and to *actuality*. On the other hand, the practical and mimetic acquisition of language and its constant semantic re-honing seem to have a strong relation to *locality*- as spacio-temporal context. In contrast with the influential ‘recursion thesis’, James Hurford persuasively argues that what distinguishes human language from chimp language is the proliferation of words in human languages which ‘contain tens of thousands of arbitrary learned symbols’ (Hurford 2004, p. 551). It is the abundance of these discrete verbal or phrasal elements, merging or linking in self-concatenation, according to embedded rules, which form human speech. Their mimetic acquisition, from other human beings, in given contexts of application and association, is a hyperbolic, species-wide education in human particularity as social reality: infinitely repeated, in every utterance, as mind, sound, tone, inflection—or script—come together in ever new ways, but always in actual form, at a specific time and place. Even within its innumerable connections and coalescences, each word has particularity: within the *lexis* of a language, it is or has *haecceitas*. In genus *Homo*, mimetic language acquisition and its continuing heuristic application is a species-wide training in co-locating with others, through our particularity, and so, by implication, in developing consciousness of a shared world.

This kind of physicalist reading of language proposed by Clark and Hurford places human language at the centre of the *locality*-*actuality* dyad and so also, in principle, at the centre of our human niche construction. But precisely how might such a linguistic niche construction work? We cannot approach this question without acknowledging the difficulty that our capacity for bonding (HC) draws upon the ancient motor system, while the ‘semantic system’ of our ALC sits in the much more recent expanded neocortex. Language and social cognition diverge in the fact that the latter system, which forms the bonding ground that animates HC, is early in our evolutionary history and is widely shared, though to varying degrees, with creatures other than ourselves. ALC is late, however, and perhaps very late, only being attested in the archaeological record from some 15,000 years ago. As human subject, we experience these as if they were co-eval in us, but there are in fact unmistakable longitudinal and anatomical distances between them. If we are to be able to develop the outline of a critical account of the role of language in niche construction, then the significance of these distances will need to be clarified. Section Two below sets out the nature of our—recently discovered—early social cognition, highlighting some of the questions raised by the different kinds of terminology (both scientific and philosophical) which have been or may be used. In Section Three we shall look more closely at the nature of the structural relation which obtains between early social cognition and late developing ALC, and shall place this within EES as an internal form of niche construction.

## Social cognition

### Social cognition and the internal ‘in-between’

‘Social cognition’, which is closely linked with ‘Autism Studies’, is the ‘study of information processing in a social setting’ (Frith 2008, p. 2033). As such, it approximates to being the study of the internal structure of HC. The ‘cognitive system’ at the centre of our social cognition, is defined by Vogeley in the following way:

The point of departure is a fundamental understanding of cognitive processes: a cognitive system is a system which in its reaction to environmental stimuli shows some degree of flexibility, a factor that is made possible by internal information processing (Vogeley et al. 2014, p. 16).^4^

The interrelational communication which takes place between bodies in ‘online’ social cognition (i.e., in the actuality of the face-to-face) is itself both ‘organic’ and ‘intelligent’ in that our responses are interactive, guided by the information about each other which is constantly being exchanged. This has been summarized in the following terms:

When we interact with another person, our brains and bodies are no longer isolated, but immersed in an environment with the other person, in which we become a coupled unit through a continuous moment-to-moment mutual adaptation of our own actions and the actions of the other (Konvalinka and Roepstorff 2012, p. 2).

These multiple reflex interactions occur at speeds well below the threshold of conscious perception, but communicate as a sense of ‘rapport’ (Tickle-Degnen and Rosenthal 1990). As ‘complex, multi-layered, self-organizing’, they sit within the early motor system, involving sets of mutual responses ranging from eye movement, facial expression, posture and gesture to the synchrony of brain waves, breathing and pulse: a subtle and pervasive ‘alignment of behaviour’ which includes ‘synergies, co-ordination and phase attraction’ (di Paolo and de Jaegher 2012, p. 1; Schilbach et al. 2013). The mutual ‘costly signalling’ of the ‘cognitive system’ risks that any deviation will be spotted by the other, but the open engagement fosters mutual discernment and, further, contributes prosocially to the building of cooperative community.

The correspondence of the social ‘cognitive system’ with language also appears in the intensive reflexivity of the motor responses which, though pre-thematic, are self-monitoring and monitoring of the other, as well as any third party observers who may be present, during this process of ‘internal information processing’ (Vogeley et al. 2014; Schilbach et al. 2013). There is also a complex engagement with the other which recruits the medial prefrontal cortex in processes of evaluation, which may need to integrate different sources of knowledge: a first-hand knowledge of the other gained in the moment and a second-hand, associative knowledge acquired from other sources. These two modes of knowledge may well be in conflict with one another, and can place considerable stress upon the evaluative system (Seitz et al. 2009; Kuzmanovich et al. 2011). Within the ‘cognitive system’, the evaluative protocols of one are densely exposed to the evaluative protocols of the other, and so such a system has to be understood as a form of pre-thematic reflexivity which is extensively conditioned by an environment of interactive, physical-social complexity. Empathy, affectivity and evaluation all combine with high levels of pre-thematic reflexivity in what di Paulo and de Jaegher call ‘participatory sense-making’ of the human other (2012, p. 2).

### Philosophy, language and the ‘in-between’

The encounter with our own ‘cognitive system’ at the heart of our social cognition is a significant event in the history of our scientific self-understanding as social animal. It establishes the structure of the embeddedness of our social judgments, and delineates the pre-thematic environment within which ALC and our linguistic reflexivity function. But precisely because social cognition is so fundamental to the human, and to our existence in the world as material form, it presents a challenge to our naming, or self-naming, in its ambiguous ontology. Do we see here the ‘self’, or two ‘selves’, or alternatively, do we see neither? Which comes first? This is ultimately a question about where the boundary between self and self, self and world, lies. From a humanities perspective we might say that a human life is itself a way of answering this question, pragmatically, over time, through values, insights, relationships, and the embodied practices of living. But the life we live is our own, and so, naturally, each of us will have our own point of view on this question. This does not mean to say however that there is not something important at stake in how we answer it. This is not about relativism, but about the recognition that some human questions are too deep and too embedded within the complex real, for us to achieve clear cut answers to them. The right naming of the ‘in-between’ is just such a question; and yet still we must name it, even if, in doing so, science also becomes philosophy.

The strangeness of the naming of the ‘cognitive-system’ in the technical literature is already indicative of a philosophical nuancing. The term ‘cognitive-system’ is deflationary, while the ‘in-between’ of this section header, being prepositional, tells us *where* it is but not *what* it is. The same is the case with ‘non-local mind’ (Chatel-Goldman et al. 2013). To call it ‘rapport’ is to determine a physical phenomenon as psychological (Tickle-Degnen and Rosenthal 1990). One research team reaches for metaphor with ‘dark matter’, on account of the unparalleled, organic, informational density of ‘second person neuroscience’ and its online data (Schilbach et al. 2013). Others recognize the acute anthropological issue which is in play here: do we trace already in this motor-reflex ‘cognitive system’ the origins of the cogito, with its subject-object distinction (according to Theory-Theory), or do we see here rather the live, interrelational, mimetic matrix of physical reflexes from which consciousness itself first arises (and which accords with Simulation-Theory) (Chatel-Goldman et al. 2013)? The term ‘enactivism’ prioritizes the latter, understanding the dense, interactive, self-organising structures of our social cognition to be themselves the ‘enactment of a world’ (cf. di Paulo and de Jaegher 2012; Hutto and Myin 2012; Kiverstein and Miller 2015). Theory–Theory defines the ‘in-between’ as the root of a cogito learning to infer the existence of another person from within interactive physical processes (in a classically Husserlian phenomenological mode), while for Simulation-Theory it is rather the root of ourselves as self-aware subject, emergent within a reciprocal mimesis that is foundationally relational. The implication in the latter may be that we now have to come to terms, as ALC, with an understanding that our own ‘prehistory’ or pre-thematic grounding and embodied belonging in the world, occurs prior to the subject-object divide. Moreover, the language of the ‘enactivist’ turn allows us to see what the implications of this naming might be. If it is the *world* which is enacted in us, and between us, rather than we who are autonomous subjects in the world, at this fundamental level, then we can also name it ‘world *in us*’ (or ‘between us’; or ‘in us and between us’), or indeed ‘the universe in us’ (activating the link between brain and quantum theory; see Fisher 2015 on quantum neural processing), or we might choose to call it ‘planet earth in us’ (inviting the articulation of a potentially planetary, distinctively eco-political, human identity). Indeed, with an eye on niche construction, and our evolutionary history, we can also call it ‘place’ or ‘internal place’, as the ultimate source of our distinctively linguistic consciousness. On account of its fertile ambiguity, how we choose to name our own foundation in the world, can have significant implications for how we make sense of it, and so also for how we practice our fundamental sociality as primary personal and social orientation in the world.

#### Scienticity, phenomenology and hermeneutics in Paul Ricoeur

An extended discussion of ‘scienticity’ and the ‘limits of consciousness’ by the hermeneutical philosopher Paul Ricoeur clarifies what is at stake in the naming of our fundamental belonging to the world. This discussion pervades his work and is set out succinctly in ‘Phenomenology and Hermeneutics’ (Ricoeur 1975, pp. 87–93), with implications down to the present day (e.g., Romano 2015). Ricoeur sets out the grounds for his ‘phenomenological hermeneutics’ by critiquing Husserlian phenomenology, with its presupposition that it is with the self-positing cogito, as source of the subject-object divide, that philosophy must begin. Ricoeur argues that consciousness is already *in medias res* of meaning-making processes, and precedes the subject-object divide as a ‘relation of inclusion’. Ricoeur captures this structure in the term ‘belonging-to’, indicating our primary existence in the world, as ‘the ontological condition […] thanks to which the inquirer shares in the thing he questions’ (Ricoeur 1975, pp. 88–89). Ricoeur holds that the decision to begin with consciousness at the point of origin of the subject-object divide, is itself a result of the cogito’s exercise of its own ‘self-responsibility’, to designate a point of departure with no foundation beyond purely its own act of doing so (Ricoeur 1975, p. 88). He opposes to this a self-awareness which acknowledges that it is already preceded by a ‘belonging to the world’, in the unity that is prior to the appearance of the cogito with its subject-object divide. For Ricoeur, this pre-thematic ‘belonging to’ offers a better grounding for the ‘scienticity’ which Husserl seeks than does Husserl’s own claim that consciousness itself can provide that foundation. Ricoeur develops his argument for scienticity in terms of the need for a properly scientific ‘critique of ideologies’ which can lay bare the underlying presuppositions that shape the intentionalities of the subject in the world. The philosopher is arguing here for a new form of self-critique, which recognizes the extent to which we ourselves are material form, in a way that matches our scientific and technological knowledge of the material world around us: ‘the critique of ideologies and of psychoanalysis give us today a way to add to the critique of the object a critique of the subject’ (Ricoeur 1975, pp. 89–91).

In Ricoeur’s view, it is the multiple processes of pre-thematic, pre-linguistic ‘participative sense-making’ (di Paolo and de Jaegher 2012, p. 2) which offer a real foundation for scientific knowledge of the self. Accordingly, he draws the important conclusion that ‘consciousness has its meaning beyond itself’ (Ricoeur 1975, p. 94). Ricoeur differs from social cognition however in that while he knows that the processes of interpretation are most primary in the live conversation of the vis-á-vis, the ‘dialogic relationship’ is nevertheless too narrowly contained ‘to cover the field of explication’. Ricoeur holds that for philosophy to be critical, there has to be both proximity to what is real about ourselves but also a degree of distance which allows critical evaluation. Therefore Ricoeur does not find this ‘distanciation’ of proximity and distance, as the ground of philosophy, in face-to-face encounter, but finds it first in the language of texts which are already at a distance from the live present, or impossible immediacy, of face-to-face conversation. For Ricoeur, it is in the capacity of texts—at a remove from their authors—to refer to a world, that different modes of human belonging to the world are disclosed to us as readers, and we have the possibility of developing a foundational, comparative self-understanding. On this hermeneutical basis, it becomes possible for us properly to critique our own unreflected and prejudicial modes of living and acting in the world. The world-disclosing text serves as the foundation for a properly critical and fundamental philosophy of the self, according to this understanding of our belonging to the world.

At the heart of Ricoeur’s hermeneutics then, as both hermeneutical and phenomenological, there are two distinct but related principles. The first is ‘consciousness has its meaning beyond itself’ (Ricoeur 1975, p. 94) (i.e., we cannot just start with consciousness), and the second is ‘*Sprachlichkeit* permeates every experience, it is not however with the *Sprachlichkeit* that a hermeneutical philosophy must begin. It is first necessary to say what is brought to language’ (i.e., we cannot just start with language) (Ricoeur 1975, p. 98). Both of these principles are relevant for a new turn in phenomenological hermeneutics as a response to, and integrative engagement with, the sciences of HC. Moreover, a phenomenological hermeneutics which takes as its point of departure the ‘appearance’ of technical, measurement-based fMRI data, indexing discrete brain activation in present human social interaction, is representationally and so also hermeneutically at a far greater distance from the live phenomenon of face-to-face conversation than are natural language texts. The possibility of a more pronounced and therefore potentially more productive relation of distance and proximity, as the ground of a philosophy of the self in the world, comes into view here therefore. An enhanced dialectic of distance and proximity, over and above Ricoeur’s concern primarily with written texts, may both call for and enable a more extensive philosophical integration of fundamental scientific sources of knowledge about the self, including our enriched human subjectivity where we are, in Phillip Sloan’s phrase, ‘existentially existent, conscious and self-reflexive beings’ (Sloan 2015, p. 250).^5^ But paramountly what we are investigating here is the possibility that the identification of a new locus and intensity of Ricoeur’s ‘distance-proximity’ dialectic may cast light on human evolution and the acquisition of our advanced human language as a form of ‘internal niche construction’. This in turn may tell us more about what is distinctive to our human evolution in ways that can potentially help us better to understand the internal structure of co-operative human social behaviour.

## Internal niche construction

### Language and the production of ‘social place’

We have seen that the question of what the new neuroscience of social cognition can tell us about ourselves philosophically requires two levels of refinement. The first rests within Husserlian–Heideggerian phenomenology and asks: ‘what do these signs generated on fMRI scanners *mean*, in which something is made visible about ourselves which was previously hidden?’. The second refinement sits within Ricoeur’s hermeneutical critique of the classical phenomenological method with the latter’s insistence on beginning with the subject-object divide, and its refusal of the pre-linguistic. Ricoeur insists rather that ‘consciousness has its meaning beyond itself’ (Ricoeur 1975, p. 94). With Ricoeur, we can now ask: ‘what do these signs tell us about what is *brought to* language (or linguistic consciousness)?’ If we resist our long habit of thinking of ALC as ‘bringing’ rather than ‘being brought to’, then this issue of our ‘belonging-to’, or how we are in the world as ALC, becomes key. For Ricoeur it is only by addressing this question that we can develop a properly self-critical perspective on how we *live* together in the world, in terms both of what we believe and our practices of reasoning. For Ricoeur, this question first appears in a critical form not when we consider the live present of conversation, but rather when we reflect on how ‘belonging-to’ is reproduced in literary texts. Here for the first time we find sufficient ‘distanciation’ or ‘proximity and difference’ to support philosophical self-critique. Our point however is that the communication of our ‘belonging-to’ in the neuroscience of social cognition seems more comprehensively to represent this structure of ‘distanciation’, and so potentially offers the possibility of a more far-reaching form of philosophical self-critique. Now the self, as ALC, encounters in signs what ALC itself pre-thematically *is*.

What we see here, more powerfully than in the case of texts, is a structural relation which admits of clear critical analysis. The relation between late developing, self-aware ALC and its early pre-thematic ground is a *dynamic* one on account of the dual inheritance of language. On the one hand, language is the product of the social cognition system, with its embodied communicative interrelationality and bonding. But on the other, it is the child of tool use, as an outward-looking way of shaping the world beyond the interrelation of bodies, through the projection and realization of our intentionalities. These are distinct in that each displays a different orientation to the world. The former allows an internal orientation to world, since the ‘in-between’ or social cognition system is self-organizing and can be called ‘world in us’. Here language can itself be the conduit for, and become expressive of, that social world within us. ALC can itself image that ancient communicative system since, by virtue of the materiality of the sign, language remains rooted in the internal landscapes of our embodied social cognition. In this case then it is the activation of language *towards the other*, in a way that applies social reasoning with and for the other, which constitutes the internal orientation of ALC to world, through the convergence of our social reasoning with the ‘in-between’ within us. But language is a product of tool use too, which brings the second, external orientation of ALC to world. Here the capacity of words to function as cognitive tools for linguistic classification and control is uppermost, as we seek to understand and manage our environment as the world around us. But words can also exhibit a capacity to function as ‘social tools’, whereby we seek to manage those other human beings with whom we share and shape our human niche.

What we are observing here are two related but distinct forms of niche construction through language, in two distinct dynamic forms of action, showing on the one hand an internal orientation of ALC into the human body through conversational recognition of the human other and, on the other hand, an external orientation of managing the world and shaping it in ways that allow us to be at home in it. To the extent that external niche construction in human beings involves HC, we can say that internal niche construction is *presupposed* in the viability of our species, as the basis of HC. But how does internal niche construction occur, what kind of work is involved in it, and what does this work produce?

At the centre of internal niche construction is the phenomenon we are calling ‘social reasoning’. This is the personal and dynamic *judgment* which is affectively, empathetically, volitionally and conscientiously structured towards the other, in their co-locatedness (Janz 2008; Diamond 2003; Cockburn 1990). It is sometimes discussed as compassionate reasoning (Nussbaum 1996; Davies 2001). In its power of judgment for and with the other, social reasoning shows an engagement with the other and a recognition of their own ‘belonging-to’ in the world or perspective on the world, which is convergent with the ‘participatory sense-making’ or interactive cognitive structure of our social cognition (di Paolo and de Jaegher 2012, p. 2). Significantly, evaluation plays an important role too in our pre-thematic social cognition, since diverse channels of knowledge need to be reconciled in processes of personal, cognitive interaction which, despite their pre-thematic nature, are certainly to be understood as a profound and intimate engagement of the human body with complexity: the complexity of the human other. High functioning evaluation which is associated with the medial prefrontal cortex, is a central part of the social cognition system, and sits within a matrix of affectivity, empathy and reflexivity. We can note too that in conversation, language itself becomes a heuristic tool for consciously discerning the other, in a way that parallels the function of face and body for the pre-thematic social cognition system. Live conversation shares the recursivity of social cognition in its progressive ‘messaging’ or ‘internal information processing’, and parallels social cognition in its ‘costly signalling’ or far-reaching transparency to the other (Vogeley et al. 2014; Clark 1996).

Internal niche construction can be defined as this communicative convergence between ALC and the social cognition system, in live conversation with another. This reaches its highest resolution in our social judgments, arising from our social, other-orientated reasoning. Judgments, involving discernment and decision, mark the point of our self-positioning. Social judgments for and with the other—which are inevitably judgments in complexity—become the form of our own self-positioning which is convergent with the recognition of the other which is entailed in our social cognition system.^6^

But what kind of work is done here? The work that is done in internal niche construction concerns resistance: resistance to the naturally controlling aspects of language by which we ‘arm’ ourselves. Internal niche construction requires an openness to the other, which is a form of deliberate linguistic ‘dis-arming’. Hospitality, responsivity and openness are the conditions of internal niche construction, and ‘social reasoning’ does not entail that we judge the other. It is rather how we come to judgment for and with the other. But consciousness, as Bernard Baars argues, is a ‘global workspace’ or system which facilitates coordinated decision-making in an advanced brain which is tuned to the world’s complexity and which might otherwise be incapable of making good, quick decisions (Baars 1997; Frith 2007). ALC is an integrative organ of judgment, ordering and hierarchalization, running on a platform of predictive perception, which Andy Clark calls ‘surfing uncertainty’ (Clark 2016). Its most challenging moments are those which require maximum levels of integration across different brain areas, as in the case of our moral or social decision-making (Greene 2015).

Consciousness as a system of judging and controlling significantly contrasts with the receptivity and responsivity of our ‘social reasoning’. The ALC we associate with HC is constantly confronted with, and potentially disrupted by, the unparalleled complexity of the human other. While ALC can reduce the complexity of the world by coming to judgment in a controlling way, through appropriate self-interest and instrumental reasoning (reflecting the functionality of the tool set), the attempt to reduce the personal complexity of the human other by the imposition of our own needs and perspectives will be likely to undermine friendships and alliances. In small scale, non-hierarchical, highly cooperative societies, the dominance of such reasoning seems unthinkable. But how do we explain the emergence of a system by which consciousness, which normally seeks to reduce complexity through distancing and control, now comes to judgment in ways which appear to embrace complexity—of the proximate human other—bringing about the renunciation of instrumentalization and control?

Evolutionary theory and niche construction can offer a context within which such co-locatedness or mutual recognition and solidarity of an altruistic HC might make good sense, despite the primary functionality of ALC as an instrument of de-complexifying control. The complexity of the world is reduced by sustained controlling mechanisms through cognition and action; but it can also be managed through an entirely different resource, which we can term ‘option for the other’. Such an option signals significant bonding. While it fails to reduce complexity on the one hand, it nevertheless increases the human resource for dealing with the world’s complexity on the other. It makes sense that a highly intelligent creature who is tuned to the world’s complexity, managing it through astute ‘tool use’, including the incisive use of language, should be skilled too in multiplying the human resource, through capacities for shared judgment and the practical recognition that we are differently located in a shared world. Indeed, we can see the social cognition system as an inherited social embodiment reflecting the history of generations of genus *Homo*, since the shortening of birth intervals in *Homo erectus* which led to a significant increase in altruistic collaboration and social cohesion, from at least 1.8 million years ago (Aiello and Key 2002).

But if there is work in internal niche construction at the point where we choose our words, resisting the natural controlling mechanisms of ALC, then what does this work produce? Firstly, it produces a degree of harmony within our own unity of mind and body, as ALC converges with the sophisticated symmetries of our social cognition. This means also that it produces a degree of human integration, as mind finds itself ‘at home’ in the body, and so also in the world, from the perspective of an internal orientation. We can call its primary product ‘social place’. This suggests an ecology of responsiveness and co-locatedness, which saturates the proximate environment. ‘Social place’ signals the ecstatic objectification of the sociality of the interfacial, where ALC is ‘disarmed’ and becomes the image of the ‘in-between’. ‘Social place’ becomes the ground of HC where it is constantly renewed, not least through the many forms of linguistic ‘disarming’ such as dance (Noy et al. 2015), music (Hove and Risen 2009) and performance (Konvalinka et al. 2011) which repeat the mimetic and rhythmic structures of our social cognition outside the framework of semantic structures of teleological control. A healthy ALC repeatedly returns through its own ‘disarming’ to its basal, social belonging to the world, in the generative production of ‘social place’.

#### Hyper-place

‘Social place’ is produced within interfacial community, where ALC yields to the self-communicating, pre-thematic ‘language’ of the human body which is its ground. Here language is ‘disarmed’ through the performative reception of its own social, pre-thematic ground of rhythm and synchrony. Strong, symmetrical convergence occurs between ALC and its ground in ‘social reasoning’, with its other-orientated judgment within complexity. This allows the transformation of the internalized materiality of the sign from within: its re-assimilation as tool back into its ground in the interrelationality of social cognition, in the production of ‘social place’ as a *tertium quid*. In evolutionary terms, this is the internal socialisation or re-socialisation of late-comer language.

But this brings us before an important question recently raised by Wendy James (James 2017). If our understanding of ‘sociality’ is to be properly distinctive in the context of human evolution, then it will need to find a place for ‘religion’ which, by common agreement, cannot straightforwardly be applied to non-human animals. Religion is complex, especially in its definitions, and not to be dealt with in the conclusion to a short paper. But if we define religion as world religion, which is transgenerational and trans-regional, then we can be confident that we are dealing with something that shapes our global environments. The term ‘hyper-place’ signals the sense of visceral belonging which holds together international religious community identities and which may have echoes too in the inclusive identities of the great powers such as China, US, India, Russia and EU (Asad 2003). This is visceral belonging beyond the face-to-face. ‘Social place’ may be something that is shared with non-human animals (primates and some bird species come to mind), but ‘hyper-place’ is an exclusively human phenomenon. What kind of work in niche construction might produce ‘hyper-place’?

We shall argue here that the ‘hyper-place’ we associate with world religions reflects a particular kind of productive work at the point of interaction of both internal and external niche construction. Internal niche construction, with its social and ethical nature, shows a power of convergence through ‘social reasoning’. World religions do generally exhibit features of this convergence. Typically, they operate with strong ethical imperatives, though contrary to the view that these reduce ethical reflection on account of their heteronomy, they tend rather to be generic (e.g., the Christian ‘Love your neighbour as yourself’ or the Buddhist concept of ‘skilful’ living). The religious, ethical imperatives thus demand repeated and often difficult, unpredictable contextual applications, constantly bringing the subject back to encounter with complexity. If not generic, then they are legalistic and multiple, and so, once again, require interpretative evaluation (e.g., as reflected in the legal codes of Islam or Judaism). We can see the weight of religious imperatives and the normativity of the religious ethical community as enhancing the ‘social reasoning’ and ‘option for the other’ which is productive of ‘social place’.

Similarly, we can see parallels in the ritualized language of the religious community which typically displays de-controlling postures based in the acknowledgement of supernatural powers and ultimate ends. Religious rituals can give extensive emphasis to the materiality of words—through cantillation, calligraphy, dance, singing, illustration—preferring evocation and symbol to the accurate and detailed language use we associate with cognitive control. Of course, control reappears *within* the individual communities, through religious hierarchalization and claims to responsibility for ‘tradition’, and through precisely formulated normative beliefs. In ritual settings, the cultural enhancement of affectivity, mimetic structures of dance, gesture, and song, the display and following of religious exemplars, and the thematization of compassion (in the naming of God in abrahamic religions) combine with ethical precepts in what seems to be a communitarian and cultural form of ‘social reasoning’.

But where do we find external niche construction in religion, beyond the evident visible effects of religious community and life? The point has been well made recently by Ingold and Fuentes that the imagination plays a critical role here (Ingold 2013; Fuentes 2014). Religious communities inherit potent ways of imagining what the world is like, beyond empirical reality: they inherit a different ‘way of seeing’. But any references to the role of imagination in religion need to be circumspect in the sense that a deep tension is discernible throughout the modern period between reason and imagination which we can associate historically with the rise of Romanticism in response to Enlightenment. In their origins, the world religions predate the Enlightenment however. In the case of Christianity, in the Western world, we can see that one of the effects of the Enlightenment was to change the *realism* of belief in the Judaeo-Christian cosmology of the pre-Copernican, finite universe (familiar to us from medieval maps) to a set of *metaphorical* beliefs which are superimposed on a scientifically verified account of the world. These metaphorical beliefs appear as imaginative and creative ways of asserting the centrality of the human person in the world order, and the possibility of our ultimate ‘at-homeness’ as creature in the Creator’s universe. But they are not as such realist beliefs. Prior to the rise of the modern scientific method however, belief in the invisible and heavenly dimensions of the universe was not an imaginative assertion but rather the envisaging and representation of what the world was thought to be like beyond our present sense perceptions. In its own time, this was realist belief (Davies 2004). Furthermore, it supported a *cosmic* level of human niche construction. The Creator’s universe was understood to be our niche—where we can ultimately be ‘at home’—and the religious way of life was the path to be taken to that unending and cosmic form of belonging.

From one perspective, it makes sense to assert that what we are calling ‘hyper-place’ may simply reflect the sense of belonging together across boundaries that is engendered through the shared practices and ethics of world-wide religious communities. This is an argument from Theory of mind which asserts that we are more likely to trust those whose inner life is present to us by analogy with our own inner life, as developed by those beliefs and practices which we share across boundaries. But there may be more to the bonding that animates the prosociality of the world religions than this, especially in the light of the very strong account of our belonging to the world, through the ‘in-between’, which social cognition gives us.

From the perspective of niche construction theory then there may be another approach to explaining the sense of shared consciousness which arises in world religions. Internal niche construction involves convergence between ALC and the social cognition system. This is effected by an embrace of the body’s own ‘option for the other’ through the reception and recognition of an actual, complex human other by ALC. The internal orientation of internal niche construction is one of the convergence and harmonization of ALC with the ‘in-between’, through ‘social reasoning’. Our openness to the actual human other, in their complexity, manifests also as our openness to the presence of world within us, in the ‘in-between’, through the dynamic of convergence. We are in the world as the world exists within us, to the extent that we move towards the other who comes to meet us. In each case, it is the radicality of our openness to the complexity of the world, in the other and within us, that predominates.

But why should this engender the sense of a social unity, rooted in a shared world, which suggests a very deep apprehension of community across cultural, geographical and indeed historical distance? The answer may lie in the nature of the external orientation of external niche construction through the imagination in world religions. Once we add the cosmological imagery of religious belief to a convergence with the world within us through the ‘option for the other’, then a possible symmetry opens up between the radical openness of our internal niche construction on the one hand, and an ultimate openness to the world around us, through imaginative realization, on the other. If we already *experience* oneness with world through the ‘option for the other’ and our internal convergence with world in us, then why might the reality of the former not be *represented* in the latter? What happens if the same religious framework which enhances the ‘option for the other’ as a principle of living, also shapes the subject’s openness to the world as cosmologically conceived? Perhaps an internal oneness with the world, would then appear also as a shared understanding of the world, and a common sense of being cosmologically at home in the world?

Non-reductive physicalism insists on the paradox that even though mind and matter are different, they are not separate: they form a single unified system, corresponding to Velmans’ ‘reflexive monism’ (Velmans 2009). But as we have seen, to be human is to be in the world and is to have two kinds of niche construction: one internal and the other external in orientation. In the first we encounter the world as it exists within us, and in the latter as it exists outside us. But this ‘within us’ and this ‘outside us’ are both in the world: they are both world. In a non-dualist scheme of things, the perspectival sense we have of being a separate observer of the world, is itself as much in the world as is anything else.

It is possible then that in combination, internal niche construction and external niche construction together may permit a sense of the oneness of the world which transcends the separation of our perception of it. If that is the case, then we could never find a way of explaining this experience through language or propositions. By the ‘dis-arming’ of language through ritualization and rhythmic movement, within a communitarian framework of religious imperatives and imaginings, what we are here calling internal and external niche construction may produce at their intersection ‘hyper-place’, designating a common sense of oneness arising from within the diversity of life. Perhaps Tim Ingold gets it right when he defines the ‘grammar of representation’, which he links with science, as distinct from the ‘grammar of participation’, which he links with religion, in the following terms: ‘[w]e grow into the world, as the world grows in us’ (Ingold 2013, p. 746). But what might the implications be if it is through the ‘grammar of representation’ that the ‘grammar of participation’ is discerned?

## Conclusion

In summary then, this paper sketches the outline of an interdisciplinary project or projects for developing a more critical understanding of how we construct our human niche, from the perspective of ‘internal niche construction’. This term points to the modern linguistic human subject in relation to her own ground in the social embodiment of genus *Homo*. Through the internalized materiality of the sign, this is a transformational relation, and so also potentially a productive one. Such a project may potentially have value today in highlighting the creative tensions which can exist between the self-communicating inclusivity of ‘social place’ and ‘hyper-place’ on the one hand, and between both ‘social place’ and ‘hyper-place’ and the sometimes militarized territorial boundaries that sectorize the planet on the other. This points to a contrast between the politics of deep community and the politics of the nation state. We can ask the question of what we can learn about the formation of global solidarity from world religions, and indeed about the religious deformations which occur, for instance, where the claim to limitless territory entails the eradication of compassionate ‘social place’, in the failed production of ‘hyper-place’? What does the production of ‘hyper-place’ tell us about the formation of stable, large-scale ethnic or political identities? What does it tell us about how we can enhance community through the way we read early texts? Can such archives effectively ‘produce place’ in a past cultural idiom? What might this phenomenological hermeneutics tell us about the hidden motors of social change, through the organization of space, or people, or through the power of art? And what might that tell us in turn about the humanization of our institutions and how they can be made more responsive to the way we ourselves humanize space as place? And finally are there insights here of a clinical or therapeutic kind? Can the work of a mediator or therapist be understood as a way of ‘admitting’ the bioenergy of the ‘in-between’ into the place of meeting as a moment of solidarity that can be healing? If we have already been using our social cognition intuitively in so many ways and for so long, as genus *Homo*, then it seems important that we should also take the opportunity today to understand it scientifically, and critically, and so in ways which may allow us to draw valuable lessons from our past in the construction of our future.

## Footnotes

1 In this paper we shall focus principally upon the particularity of the linguistic-literary perspective. For a study of pre-modern mystical texts as a potential contribution to extending the horizons of hermeneutical philosophy from a historical perspective, see Davies (2015).

2 In ‘Questioning the Zoological Gaze. Darwinian Epistemology and Anthropology’, Phillip Sloan skilfully deploys Erwin Strauss’ phenomenological analysis of bipedality (Sloan 2015). On phenomenology and consciousness, see also Gallagher and Zahavi (2012).

3 Chris Hatfield’s popular performance of ‘Space Oddity’ from the international space station, for instance, began a process of humanizing ‘outer space’, shaping it as our ‘niche’, and so also as the potential new domain of our intentional activity, for all who have heard him (https://www.youtube.com/watch?v=poZCINzxzrQ. Accessed 9 Jan 2016).

4 My translation.

5 There may also be a resonance here with current calls, within ‘radical enactive neuroscience’ (REN) for instance, for more networked or ‘global’ accounts of the unity of cognition-emotion in human beings (Kiverstein and Miller 2015).

6 Robert Kane’s analytical philosophical position on moral actions shows significant similarities here. For Kane, ‘indeterminacy’ serves as complexity, and ‘self-forming actions’ as convergent ‘social reasoning’ (Kane 2009).
